# Superior mesenteric embolus accompanied by renal infarction associated with atrial fibrillation successfully treated by anticoagulants: A CARE-compliant case report

**DOI:** 10.1097/MD.0000000000046740

**Published:** 2025-12-19

**Authors:** Pei-Hsien Lin, Chih-Yu Liang, Pin-Yan Huang

**Affiliations:** a Department of Emergency Medicine, E-Da Hospital, I-Shou University, Kaohsiung, Taiwan; b School of Medicine, College of Medicine, I-Shou University, Kaohsiung, Taiwan.

**Keywords:** acute abdomen, acute mesenteric ischemia, anticoagulation, atrial fibrillation, case report

## Abstract

**Introduction:**

Occlusive arterial acute mesenteric ischemia resulting from either thrombosis or embolism is a major cause of superior mesenteric ischemia, which is an emergency condition associated with a high rate of mortality. This report aimed at discussing the applicability of anticoagulants alone as a conservative treatment strategy for patients with superior mesenteric artery (SMA) embolism.

**Patient concerns:**

A 70-year-old woman with a history of hypertension and dyslipidemia presented to our emergency department with persistent moderate-to-severe lower abdominal dull pain for three hours. Her vital signs and initial laboratory studies were normal.

**Diagnosis:**

An enhanced abdominal computed tomographic scan revealed a filling defect in the SMA and an infarcted right kidney suggestive of a diagnosis of SMA embolism. An electrocardiogram showed atrial fibrillation. Subsequent blood sampling demonstrated an elevated D-dimer level at 6.08 mg/L.

**Interventions:**

Surgery was not indicated due to the absence of bowel necrosis or overt peritonitis. The patient was admitted to the cardiology ward, where anticoagulation therapy with a 5-day course of subcutaneous enoxaparin was started, followed by switching to oral edoxaban.

**Outcomes:**

The patient was discharged uneventfully 1 week after admission.

**Conclusion:**

Our unexpected discovery of a case of SMA embolism complicated with right renal infarction underscored the need for vigilance in identifying the condition through imaging studies, pinpointing the potential contributor (e.g., atrial fibrillation), and implementing appropriate treatment. Our case underscores the importance of anticoagulation once the diagnosis is made and the efficacy of conservative treatment in selected patients without signs of systemic inflammation or peritonitis.

## 1. Introduction

Occlusive acute arterial mesenteric ischemia, which can be attributed to either thrombosis or embolism, is an emergency condition associated with a short-term mortality rate of 51.8%^[1]^ and an overall fatality rate of up to 93%.^[2]^ The incidence was 8.6/100,000 person years with a sharp increase with age.^[2]^ The reported incidence of embolism is slightly higher than that of thrombosis in a ratio of 1.4.^[3]^ The occlusion can originate from either in situ thrombosis, mainly in individuals with underlying atherosclerosis, or an embolus, such as in patients with atrial fibrillation.^[4]^

In particular, atrial fibrillation was noted in up to 28% to 79% of patients diagnosed with arterial embolism.^[5,6]^ The therapeutic approaches include medications, electrical cardioversion, as well as catheter or surgical ablation.^[7]^ Catheter ablation, in experienced hands,^[8]^ has been reported to be superior to medications in the treatment of atrial fibrillation in terms of improvements in cardiac function and quality of life in patients with heart failure.^[9]^

Despite the rarity of occlusive acute arterial mesenteric ischemia, prompt diagnosis and treatment are essential to patient survival because of the high associated mortality rate.^[2,4]^ Here we report a case of superior mesenteric arterial occlusion with renal infarction accompanied by atrial fibrillation.

## 2. Case report

**Figure 1.**
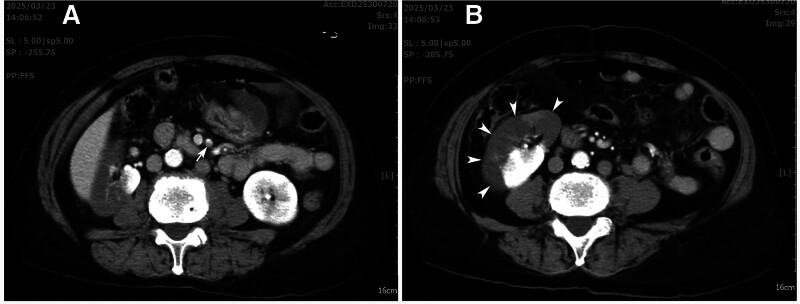
Enhanced abdominal computed tomography showing (A) a filling defect inside the superior mesenteric artery (arrow), and (B) an extensively infarcted right kidney (arrowheads).

A 70-year-old woman presented to our emergency department with persistent lower abdominal dull pain for three hours. The severity of pain was 5 on a scale of 10. She had a history of hypertension and dyslipidemia under satisfactory medical control but denied other known systemic diseases. On arriving at the emergency department, she was alert (GCS: E4M6V5), afebrile (body temperature: 36.0ºC) with stable hemodynamics (heart rate: 74 per minute; blood pressure: 131/81 mm Hg). Her respiration was smooth at a rate of 18 per minute with an arterial oxygen saturation of 99% on room air. She complained of mild nausea but denied urinary symptoms. Physical examination revealed moderate lower abdominal tenderness on palpation without rebound tenderness or muscle guarding. McBurney’s sign, Rovsing’s sign, psoas sign, and obturator sign were all negative. Laboratory studies showed a white blood cell count: 7930/mL, hemoglobin concentration: 14.6 g/dL, hematocrit: 44.1%, platelet count: 192000/mL, neutrophil: 71.2%, lymphocyte: 24.5%, alanine transaminase: 30 U/L, plasma creatinine: 1.10 mg/dL, estimated glomerular filtration rate (eGFR): 52, and a C-reactive protein: 1.35 mg/L, prothrombin time (PT): 10.1 seconds, international normalized ratio (INR): 0.98, and activated partial thromboplastin time (APTT): 28.5 seconds. Urinalysis demonstrated the presence of occult blood (50 RBCs/microliter) and trace amount of blood on microscopic examination (5–10 RBCs/HPF). Due to refractory abdominal pain after intramuscular analgesics, the patient underwent abdominal computed tomography (CT) that revealed a segmental filling defect in the superior mesenteric artery (SMA) (Fig. 1A) coupled with right renal infarction (Fig. 1B). Such unexpected findings prompted a further investigation into the coagulation profile and D-dimer concentration. While the coagulation profile was normal, the D-dimer level was elevated at 6.08 mg/L (normally < 0.55 mg/L). Electrocardiogram demonstrated atrial fibrillation. After consultation with a general surgeon, the option of surgery was declined due to the lack of imaging evidence suggestive of bowel ischemia and the absence of clinical signs of peritonitis.

She was admitted to the cardiology ward for intravenous hydration and anticoagulation with subcutaneous enoxaparin. Echocardiography showed normal ejection fraction and fractional shortening without evidence of thrombus or valvular anomaly. After 5 days of enoxaparin treatment, anticoagulant was switched to oral edoxaban at a daily dose of 60 mg, along with her previous prescriptions of rosuvastatin and propranolol for dyslipidemia and atrial fibrillation, respectively. No surgical follow-up was needed because of improvement in appetite and bowel motility. Despite transient hematuria that occurred one day after the initiation of anticoagulant treatment, the condition subsided within 48 hours after its first appearance. Because of general improvement, she was discharged to outpatient care 1 week after admission. The same oral dose of anticoagulant (i.e., edoxaben, 60 mg daily) was prescribed after discharge. She was regularly followed in the cardiology clinic and remained symptom-free despite persistent atrial fibrillation shown on electrocardiogram 1 week after discharge. Her latest blood biochemical study 3 months after discharge showed renal function (creatinine: 1.15 mg/dL, eGFR: 50) comparable to that during her first visit to the emergency department (creatinine: 1.10 mg/dL, eGFR: 52). No clinical signs suggestive of vascular occlusion or coagulopathy (e.g., bruising, gastrointestinal hemorrhage) were noted at the latest follow-up seven months after discharge. Approval from the institution review board was waived in the presence of informed consent from the patient.

## 3. Discussion

For a patient presenting with acute abdominal pain, in addition to ruptured abdominal aortic aneurysm, acute colitis, bowel obstruction, gastrointestinal perforation, diabetic ketoacidosis, and malignancy, acute mesenteric ischemia should also be ruled out as a differential diagnosis.^[10]^ Acute mesenteric ischemia is attributable to multiple conditions that can be classified into 4 categories, namely, mesenteric arterial embolism, mesenteric arterial thrombosis, mesenteric venous thrombosis, and non-occlusive mesenteric ischemia.^[11]^ Although the condition is rare, with an incidence of 12.9/100,000 person-years,^[3]^ the overall mortality rate can be over 90%.^[2]^ Occlusive arterial acute mesenteric ischemia, which accounts for the majority of the condition (approximately 65–80%), is either thrombotic or embolic in origin.^[1]^ Albeit indistinguishable on imaging studies, the two origins may be differentiated based on the clinical presentations. While an in situ thrombotic lesion tends to occur in a more proximal location of the SMA and cause more extensive ischemia, an embolus more likely occludes a more distal portion resulting in a more confined ischemic damage. In addition, the former is mainly a solitary lesion, whereas the latter may be multiple due to fragmentation of a thrombus from a distant origin. Accordingly, our patient was considered to experience an embolic condition considering the limited regions of ischemia and the presence of more than one occlusive lesion.

In spite of the highly lethal nature of occlusive arterial mesenteric ischemia, clinical diagnosis is challenging. Imaging remains the mainstay of diagnosis, as over half of the patients with this condition were not accurately diagnosed before imaging studies.^[12]^ The diagnosis, therefore, is based on exclusion with the identification of vascular impairment and bowel ischemic injury in the absence of other causes, as in our case. The optimal diagnostic tool is dynamic contrast-enhanced CT.^[12]^

Regarding treatment, the two most common approaches are endovascular and open surgical interventions. While endovascular intervention was associated with a lower 30-day mortality rate (19%) and bowel resection rate (35%) than those related to surgical treatment (43% and 70%, respectively), the former had a higher reintervention rate (33%) than the latter (3%). Overall, endovascular therapy, which has been reported to be linked to fewer renal and pulmonary complications, a shortened hospital course, and a reduced length of ICU stay, is indicated for selected patients without evidence of bowel necrosis.^[13]^ With the growing popularity of endovascular techniques, surgical interventions (i.e., laparoscopy or laparotomy) are only reserved for patients with overt peritonitis aiming at reestablishment of blood supply to the ischemic bowel and resection of nonviable tissue.^[14]^ Endovascular procedures, including percutaneous aspiration embolectomy with a guiding catheter combined with thrombolysis using recombinant tissue plasminogen activator (rtPA)^[15]^ as well as percutaneous angioplasty with stent implantation^[16]^ are currently the mainstay of treatment.

On the other hand, the efficacy of conservative treatment with anticoagulants has not been adequately addressed. Current guidelines recommend initial anticoagulation as a crucial step for improving patients’ survival following SMA occlusion.^[14]^ One of the unique findings in our case was the concomitant right renal infarction. Considering the risk of hematuria associated with the use of conventional anticoagulants (e.g., warfarin),^[17]^ a 5-day course of subcutaneous enoxaparin (i.e., a low molecular weight heparin) was prescribed for our patient. Notwithstanding the occurrence of hematuria one day after anticoagulant treatment, it was self-limited and subsided within 48 hours. Subcutaneous enoxaparin was followed by switching to oral edoxaban (i.e., one of the direct-acting oral anticoagulants, DOACs) after the acute phase as suggested by the current guidelines, which recommend treatment continuation for at least 6 months and even lifelong anticoagulation for those with underlying hypercoagulability or precipitating factors.^[14]^ Consistently, a previous case study has reported successful resolution of a left ventricular thrombus in a 60-year-old woman after one month of apixaban (a DOAC) treatment.^[18]^ Compared with warfarin, DOACs have been shown to be equally effective with a reduced risk of major bleeding in patients with nonvalvular atrial fibrillation, as in our case.^[17]^ Among all DOACs, a previous network meta-analysis showed similar safety and effectiveness of edoxaban compared to those of the others, but edoxaban was superior to rivaroxaban because of a significantly lower risk of intracranial hemorrhage and major bleeding.^[19]^ The promising outcome of our patient, who was hospitalized for 1 week without surgery, suggested the feasibility of conservative treatment with edoxaban for selected patients with acute SMA occlusion.

The current report had some limitations. First, despite the negative finding on transthoracic echocardiography, the possibility of atrial thrombosis could not be completely ruled out because the patient refused the relatively invasive procedure of transesophageal echocardiography that may provide a better visualization of cardiac structures, particularly the atrial appendages. Second, no follow-up imaging information was available in the outpatient clinic because of the lack of signs and symptoms suggestive of disease progression. Third, the results of the current report may vary with the site and degree of embolization and may not be extrapolated to all cases of SMA embolism. Further large-scale investigations comparing patient outcomes between different treatment approaches (i.e., surgery, endovascular intervention, and medication only) are warranted to optimize the therapeutic strategy for individual patients.

In conclusion, we reported a case of unexpectedly discovered SMA embolism complicated with right renal infarction successfully treated with anticoagulants. The case highlighted the importance of a high level of vigilance in identifying the condition through imaging studies pinpointing the potential contributor (i.e., atrial fibrillation in our case), as well as initiating appropriate anticoagulant treatment. Our case not only highlights the importance of early imaging in suspected mesenteric ischemia and anticoagulation regardless of the need for surgery but also underscores the feasibility of conservative treatment for selected patients without signs of systemic inflammation or peritonitis.

## Author contributions

**Conceptualization:** Pei-Hsien Lin, Pin-Yan Huang.

**Investigation:** Pei-Hsien Lin.

**Project administration:** Chih-Yu Liang.

**Resources:** Chih-Yu Liang.

**Supervision:** Chih-Yu Liang, Pin-Yan Huang.

**Validation:** Chih-Yu Liang.

**Visualization:** Chih-Yu Liang.

**Writing – original draft:** Pei-Hsien Lin.

**Writing – review & editing:** Pei-Hsien Lin.
